# Supplementary material for: Microstructural Properties of the Cerebellar Peduncles in Children with Developmental Language Disorder
Source: bioRxiv. 2023 Jul 13:2023.07.13.548858. Preprint. [Version 1] doi: 10.1101/2023.07.13.548858 (PMC10370025; doi:10.1101/2023.07.13.548858)
Supplement: Supplement 1 [file media-1.pdf]

Supplementary Table (S1): List of tests used as part of the neuropsychological battery. The measures under the *Language* and *Memory* domains were summarized into two factors using factor analysis.

Domain	Skill	Test
Language	Receptive Grammar	Test for Reception of Grammar TROG-2; Bishop, 2003
	Expressive Grammar	Clinical Evaluation of Language Fundamentals CELF-4 Sentence recall; Semel et al., 2004
	Receptive Vocabulary	Receptive One-Word Picture Vocabulary Test ROWPVT-4; Martin and Brownell, 2011
	Expressive Vocabulary	Expressive One-Word Picture Vocabulary Test EOWPVT-4; Martin and Brownell, 2011
	Narrative Production & Comprehension	Expression, Reception and Recall of Narrative Instrument ERNNI; Bishop, 2004
	Phonological Processing	Nonword repetition; Snowling et al., 2015
Reading	Decoding & Word Reading	Test Of Word Reading Efficiency TOWRE; Torgesen et al., 1999
Memory	Short-term & Working Memory	Forward and Backward Digit Span Children's Memory Scale CMS; Cohen, 1997
	Episodic Auditory-Verbal Learning	Word lists CMS; Cohen, 1997
Motor	Oromotor Coordination	Oromotor sequences subtest of the NEPSY (A Developmental NEuroPSYchological assessment); Korkman et al. 1998
	Gross and fine Motor Dexterity	Purdue Pegboard; Tiffin, 1968
Non-verbal IQ	Visuospatial Ability	Block Design Wechsler Intelligence Scale for Children WISC IV; Wechsler, 2004
	Visual/abstract/perceptual Reasoning	Matrix Reasoning WISC IV; Wechsler, 2004
	Processing speed	Coding WISC IV; Wechsler, 2004

Supplementary Material Figure (S2): The thresholded inferior (ICP; left in red, right in blue), middle (MCP; green) and Superior (SCP; left in brown, right in yellow) cerebellar peduncles overlaid on the FSL_HCP065_FA image in a typical HSL child.

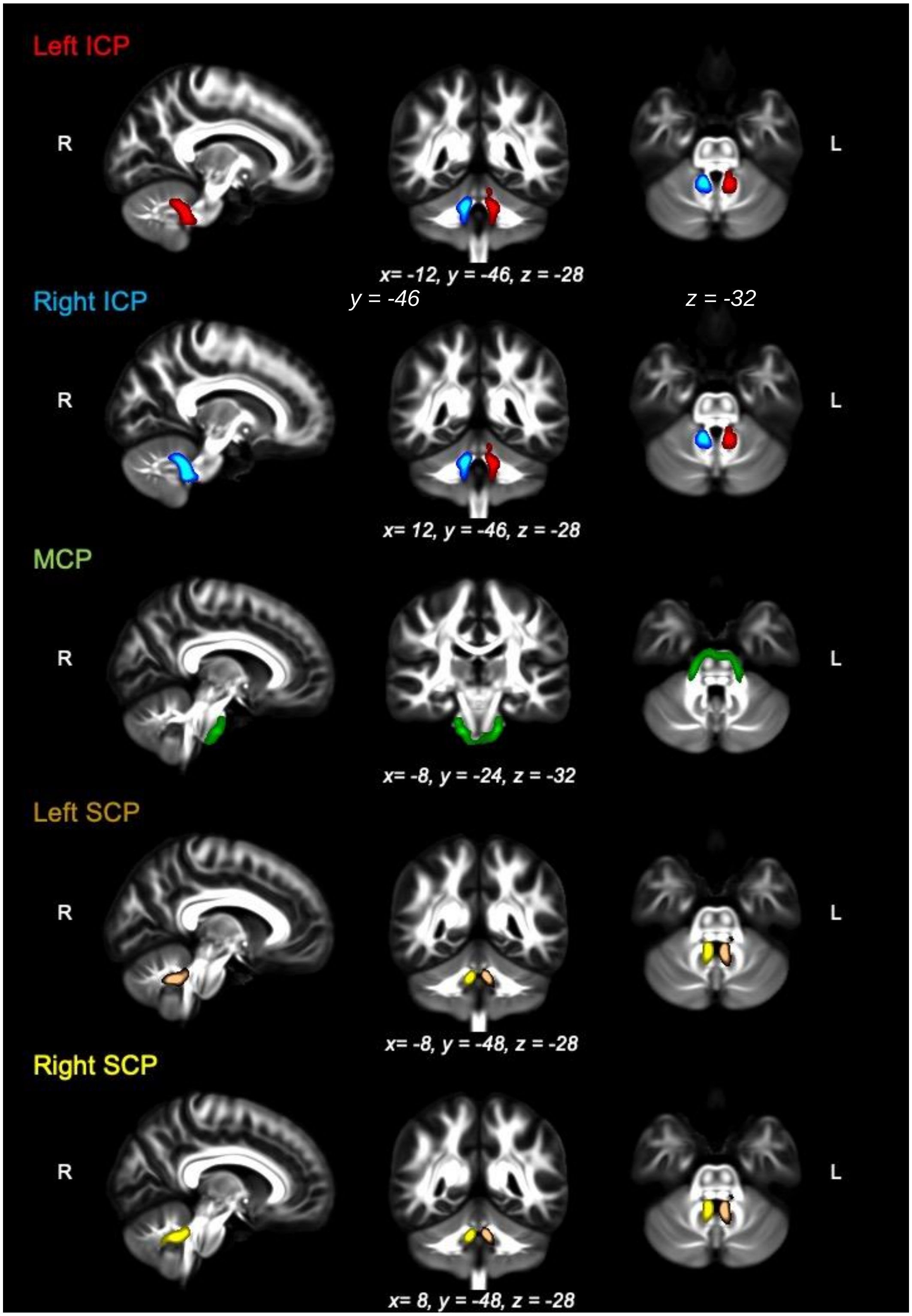

Supplementary Table (S3): Mean tract FA of cerebellar peduncles (CP) in each group

	TD (N=77)	DLD (N=54)	HSL (N=28)
**Left Inferior CP**			
Mean (SD)	0.45 (0.06)	0.42 (0.06)	0.44 (0.06)
Range (Min - Max)	0.30 - 0.56	0.27 - 0.53	0.31 - 0.56
**Right Inferior CP**			
Mean (SD)	0.47 (0.04)	0.45 (0.05)	0.46 (0.05)
Range (Min - Max)	0.33 - 0.57	0.31 - 0.55	0.33 - 0.53
**Left Superior CP**			
Mean (SD)	0.43 (0.03)	0.41 (0.03)	0.44 (0.03)
Range (Min - Max)	0.38 - 0.51	0.34 - 0.48	0.39 - 0.54
**Right Superior CP**			
Mean (SD)	0.42 (0.04)	0.41 (0.03)	0.43 (0.03)
Range (Min - Max)	0.34 - 0.51	0.34 - 0.49	0.37 - 0.48
**Middle CP**			
Mean (SD)	0.43 (0.04)	0.42 (0.04)	0.42 (0.03)
Range (Min - Max)	0.29 - 0.50	0.33 - 0.52	0.34 - 0.49

Supplementary Table (S4): Model summaries for fractional anisotropy (FA) in the inferior cerebellar peduncles (ICP) when excluding HSL group from the analysis.

	ICP Model			ICP Model including Age, Sex, Motion		
Predictor	Beta	95% CI[1]	p-value	Beta	95% CI[1]	p-value
group						
TD	—	—		—	—	
DLD	-0.09	-0.17, -0.02	0.013	-0.08	-0.16, -0.01	0.025
hemisphere						
l	—	—		—	—	
r	0.09	0.03, 0.15	0.002	0.09	0.03, 0.15	0.002
whole_brain_FA	1.6	-1.8, 5.0	0.4	1.7	-1.8, 5.1	0.3
group * hemisphere						
DLD * r	0.03	-0.05, 0.12	0.4	0.03	-0.05, 0.12	0.4
ageInYears				0.00	-0.01, 0.02	0.6
sex						
Male				—	—	
Female				0.08	0.02, 0.14	0.006
relMotion				0.11	-0.13, 0.35	0.4

[1]CI = Confidence Interval
